# Reducing Tumour Hypoxia via Oral Administration of Oxygen Nanobubbles

**DOI:** 10.1371/journal.pone.0168088

**Published:** 2016-12-30

**Authors:** Joshua Owen, Conor McEwan, Heather Nesbitt, Phurit Bovornchutichai, Raymond Averre, Mark Borden, Anthony P. McHale, John F. Callan, Eleanor Stride

**Affiliations:** 1 Oxford Institute of Biomedical Engineering, University of Oxford, Oxford, United Kingdom; 2 Biomedical Sciences Research Institute, Ulster University, Coleraine, Northern Ireland, United Kingdom; 3 Avrox Technologies Ltd. Copgrove, Harrogate, North Yorkshire, United Kingdom; 4 Department of Mechanical Engineering, University of Colorado, 1111 Engineering Drive, Boulder, CO, United States of America; University of Dundee, UNITED KINGDOM

## Abstract

Hypoxia has been shown to be a key factor inhibiting the successful treatment of solid tumours. Existing strategies for reducing hypoxia, however, have shown limited efficacy and/or adverse side effects. The aim of this study was to investigate the potential for reducing tumour hypoxia using an orally delivered suspension of surfactant-stabilised oxygen nanobubbles. Experiments were carried out in a mouse xenograft tumour model for human pancreatic cancer (BxPc-3 cells in male SCID mice). A single dose of 100 μL of oxygen saturated water, oxygen nanobubbles or argon nanobubbles was administered via gavage. Animals were sacrificed 30 minutes post-treatment (3 per group) and expression of hypoxia-inducible-factor-1α (*HIF1α*) protein measured by real time quantitative polymerase chain reaction and Western blot analysis of the excised tumour tissue. Neither the oxygen saturated water nor argon nanobubbles produced a statistically significant change in *HIF1α* expression at the transcriptional level. In contrast, a reduction of 75% and 25% in the transcriptional and translational expression of *HIF1α* respectively (p<0.001) was found for the animals receiving the oxygen nanobubbles. This magnitude of reduction has been shown in previous studies to be commensurate with an improvement in outcome with both radiation and drug-based treatments. In addition, there was a significant reduction in the expression of vascular endothelial growth factor (VEGF) in this group and corresponding increase in the expression of arrest-defective protein 1 homolog A (ARD1A).

## Introduction

Hypoxia, i.e. a reduction in dissolved oxygen concentration below physiologically normal levels, has been identified as playing a critical role in the progression of several diseases, including many types of cancer [1]. In solid tumours, it arises as a consequence of the rapid proliferation and atypical vasculature of cancerous tissue. This results in the development of areas in which oxygen demand outstrips supply [2]. Once a hypoxic environment develops within a tumour, cell populations become resistant to many conventional chemotherapeutic agents through a variety of adaptive survival mechanisms [3]. Similarly, hypoxia can have a significant influence upon the effectiveness of radiotherapy, since radiation induced damage requires the formation of reactive oxygen species, which is inhibited in the absence of oxygen [4]. Consequently hypoxia is now recognised as a key determinant of successful cancer treatment [4].

Strategies for treating hypoxia have included the development of hypoxia-selective drugs [5] and radiosensitisers such as nimorazole [6] as well as methods for directly increasing blood oxygenation, e.g. hyperbaric oxygen therapy [7], pure oxygen or carbogen breathing, ozone therapy [8], hydrogen peroxide injections [9] and administration of suspensions of oxygen carrier liquids [10]. To date, however, these approaches have delivered limited success owing to lack of proven efficacy and/or unwanted side effects [11]. Gas microbubbles, stabilised by a biocompatible shell have been in use as ultrasound contrast agents for several decades and have also been investigated as an alternative means of oxygen delivery [12]. Recent studies have demonstrated that peritoneal or intratumoral injection of oxygen-loaded microbubbles can be used to increase systemic oxygen levels [13] and substantially increase the efficacy of cancer treatment in animal models [14]. Encouragingly, no adverse side effects were observed in these studies; however, the risks associated with injecting high concentrations of microparticles [13] makes them unsuitable for intravenous administration and this limits their potential for clinical translation.

A further route that has been explored involves the delivery of oxygen in the form of a stabilised foam via the stomach. Oral administration of pharmaceuticals and other therapeutic materials has considerable advantages in terms of patient acceptability, reducing the risk of infection, cost and the quantity of material that can be delivered [15]. Frequently, however, oral administration is associated with inefficient delivery and/or poor bioavailability (*ibid*.). There are a number of reports in the literature of the use of foams or “oxygen cocktails” for the treatment of a variety of conditions [16–18], but to the best of the authors’ knowledge there has been very little research in this area in recent years. There have been several more recent studies of oxygen saturated waters for reducing recovery times for athletes, but the clinical evidence is controversial [19].

The aim of this study was to assess the potential of a further alternative method for reducing tumour hypoxia using an orally delivered suspension of sub-micrometre sized oxygen nanobubbles stabilised by a surfactant. By encapsulating the oxygen, higher concentrations and improved stability can be achieved than via direct dissolution in water. Oral delivery overcomes the risks associated with direct injection of high concentrations of microbubbles (e.g. embolism, lipid toxicity etc. [13]) and a liquid formulation should offer improved flow characteristics leading to more efficient delivery compared with foam, as well as greater patient acceptability.

## Materials and Methods

All animals employed in this study were treated humanely and in accordance with licensed procedures under the UK Animals (Scientific Procedures) Act 1986 under a UK Home Office Licence and with ethical approval from Ulster University. Anaesthesia was performed via intraperitoneal injection of Hypnorm/Hypnovel. Mice were euthanised by dislocation of the neck. None of the animals utilized for this work became ill or died prior to the experimental endpoint.

Lecithin and citric acid were purchased from Special Ingredients (Chesterfield, Derbyshire, UK). Glycyrrhizin and glycerol were purchased from Sigma Aldrich (Gillingham, Dorset, UK). Oxygen and argon gas cylinders were purchased from BOC gases (Guilford, Surrey, UK). The nanobubble suspensions were prepared from a mixture of glycyrrhizin (3 mg/ml), lecithin (3 mg/ml), citric acid (5 mg/ml) and glycerol (0.0125 ml/ml) in untreated tap water. A 250 ml volume of the mixture was stirred for 2 hours (50°C) to disperse the constituents. An aliquot of the liquid (1 ml) was transferred to a glass vial, degassed via vacuum pump and the headspace was refilled with either oxygen or argon. The vial was then sealed and mechanically agitated for 30 s. The degassing, gas replacement and shaking process was repeated three times for each sample. Oxygenated water was prepared by following the same procedure with only water.

The size distribution of the particles in the suspension was measured using an AccuSizer 780 AD single particle optical sizing system (NICOMP Particle Sizing Systems, Santa Barbara, CA) and via dynamic light scattering (Zetasizer Nano, Malvern Instruments, Malvern, Worcestershire), hereafter referred to as SPOS and DLS respectively to detect both micro and nanoscale particles. For SPOS, a 10 μl sample of each suspension was diluted in 50 mL of filtered deionised water in a flask under mild mixing for ~30s before injection into the instrument. For DLS, 20 μl of each suspension was diluted with 980 μl of filtered deionised water in a disposable cuvette which was placed in the instrument. Three measurements were made on three samples from each suspension with both methods.

Samples of the oxygen nanobubble suspension were also examined by transmission electron microscopy using the method developed by Owen et al. [20] for studying microbubbles. 5 μl of each suspension was applied to carbon film coated 300 mesh copper grids (Electron microscopy sciences), which had been ionized in a plasma cleaner (Harris Plasma) for 30 seconds. Each grid was inverted for approximately 1 minute in order to allow buoyant particles to accumulate on the grid surface. Grids were negatively stained by incubation on a 5ul drop of 2% w/v uranyl acetate for 30 seconds. They were then dried using filter paper and left for approximately 1 minute. The samples were imaged at 80kV with an FEI Tecnai T12 electron microscope and low-dose images were acquired at ~0.8μm underfocus with 15e^-^/Å^2^ on an FEI Eagle CCD camera.

A CDI blood parameter monitoring system (Terumo UK Ltd. Egham, Surrey, UK) was used to measure the oxygen content of 3 ml samples of the above suspensions over a period of 20 min. This was carried out in a closed flow loop consisting of a peristaltic pump (Gilson, Minipuls3, Cole Parmer, Hanwell UK) operating at a flow rate of 12ml/min and a length of Tygon tubing (with inner and outer diameters of 4 and 5.4 mm respectively) in to which the sensor was inserted. The measurements were repeated on further samples of the oxygen nanobubble suspension at 37°C to determine the impact of temperature.

The ability of the nanobubble suspensions to influence tumour hypoxia was examined in a mouse xenograft tumour model for human pancreatic cancer. BxPc-3 cells were maintained in RPMI-1640 medium supplemented with 10% fetal calf serum. Cells were cultured at 37°C under 5% CO_2_ in air. BxPc-3 cells (2 × 10^6^) were re-suspended in 100 μL of Matrigel (BD Biosciences, Erembodegem, Belgium) and implanted subcutaneously into the rear dorsum of male SCID mice. Cells were purchased from ATTC, LGC Standards, Teddington, UK. Independent identification was not carried out. Tumour formation occurred approximately 2 weeks after implantation and tumour measurements were taken every other day using callipers. Data from our laboratory indicate that the tumour oxygen levels for this particular model remain relatively constant between volumes of 170 mm^3^ and 260 mm^3^, reducing the likelihood of significant inter-subject variability (S1 Fig).

Once the tumours had reached an average volume of 256 mm^3^ calculated from the geometric mean diameter using the equation tumour volume = 4πR^3^/3, animals were randomly distributed into three groups as follows: (i) oxygenated water (4 animals) (ii) argon nanobubbles (4 animals) and (iii) oxygen nanobubbles (8 animals). Following induction of anaesthesia via intraperitoneal injection of Hypnorm/Hypnovel (150μl (i.p.) of a mixture of 2:1:1; PBS: Hypnorm (0.315 mg/ml fentanyl citrate and fluanisone 10mg/ml, VetaPharma Ltd, U.K.): Hypnovel (10mg/ml midazolam, Roche, UK) the oxygen partial pressure (pO_2_) of tumours was recorded using an Oxylite oxygen electrode sensor (Oxford Optronics, Oxford, UK). A fibre optic probe was inserted into a 21-gauge needle before insertion into the centre of the tumour tissue. The needle was withdrawn and the probe readings allowed to stabilise for 5 minutes. The oxygen level in the tumour was recorded every second for 20 min. 100 μL aliquots of oxygenated water, the oxygen nanobubbles or the argon nanobubbles were then administered orally via gavage and tumour oxygenation measured every second for a further 30 minutes. This period was chosen to avoid the need for re-administering anaesthesia and on the basis of the results of an initial pilot study (S2 Fig). A total of 8 repeats were conducted per group. Two separate measurements were performed on the animals receiving oxygenated water and argon nanobubbles.

In a separate experiment, a further 9 animals (n = 3/group) were treated with the relevant preparation and then sacrificed after 30 min later without measurement of tumour oxygen. The tumours were harvested by surgical excision and processed for further studies. We selected *HIF1*α as the primary physiological probe for oxygen delivery because the presence of oxygen results in its rapid degradation [21, 22]. It thus provides a direct and immediate indicator of increased tumour oxygen levels. In addition, we examined expression of vascular endothelial growth factor (*VEGF*) and arrest-defective protein 1 homolog A (*ARD1A*). To examine the expression of *HIF1*α and *VEGF* at a transcriptional level, RNA was extracted from tumours using Trizol (Invitrogen, Paisley, UK) and reverse transcribed using a first strand cDNA synthesis kit according to the manufacturer’s instructions (Roche, Welwyn Garden City, UK). Real Time Quantitative PCR (RT-Q-PCR) was undertaken using SYBR green (Fermentas, Cambridge, UK) and gene-specific primers in a Lightcycler 480 (Roche, Welwyn Garden City, UK). Using β-actin to as a reference, expression of *HIF1*α and *VEGF* was calculated using the comparative C_T_ (ΔΔCT) method.

For Western blotting analysis of *HIF1α* and *ARD1A* protein expression at a translational level, total protein was extracted using RIPA buffer (Pierce, Rockford, UK). Primary murine antibodies employed in these studies were anti-*HIF1α* (Millipore, MAB5382, 1:500), ARD1A (GeneTex, GTX125971, 1:1000), anti-β-actin (Sigma, A2228, 1:1000) and GAPDH (Cell Signaling Technology Europe BV, 2118, 1:1000). Following resolution using SDS-PAGE electrophoresis and transfer to nitrocellulose membranes, blocking of non-specific binding was carried out in 5% (w/v) bovine serum albumin diluted in 1x tris buffered saline containing 0.05% (v/v) Tween 20. Membranes were then incubated in the appropriate secondary antibody, goat anti-mouse IgG-HRP (1:10000 of the stock solution). Secondary antibodies were purchased from Santa Cruz Biotechnology, Heidelberg, Germany. Densitometry was carried out to quantify *HIF1α* and *ARD1A* protein expression using β-actin as a housekeeping reference. For statistical analyses, significance was assessed between pairs of data sets using a two tailed Student’s t-test and confirmed by ANOVA.

## Results and Discussion

**Fig 1:**
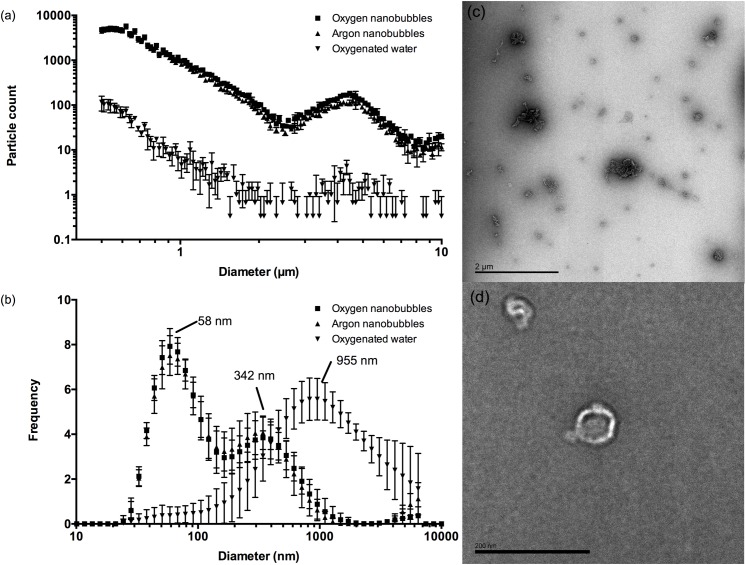
**Mean particle size distributions measured for oxygen nanobubbles, argon nanobubbles and oxygenated water by (a) SPOS and (b) DLS.** Error bars indicate the standard deviation in each measurement (n = 9; 3 readings containing 10 runs from 3 samples of each liquid). Panels (c) and (d) show transmission electron micrographs of a sample from the oxygen nanobubble suspension indicating the presence of nanoscale particles (scale bar in (c) is 2 μm, in (d) 200 nm).

Fig 1A and Table 1 show the particle size distribution as measured by SPOS in each suspension immediately following preparation. As expected, the tap water contained some particulate matter but the nanobubble suspensions contained much higher concentrations of particles. There was no measurable difference in the particle content of water before and after sparging with oxygen. The AccuSizer system is limited in terms of its ability to accurately size particles smaller than 500 nm and the fact that the maximum particle counts were at this lower limit for the oxygen and argon nanobubble suspensions indicated that they contained predominantly smaller particles. This was supported by the DLS measurements (Fig 1B) which indicated a population with a peak size of ~340nm in both nanobubble suspensions. The DLS results also indicated a sub-population of particles with mean sizes between 50 and 60 nm that were not present in water. This was supported by the TEM images (Fig 1C and 1D). Unfortunately it was not possible to determine the concentration of these nanoscale particles using the methods available. Population data were not calculated for the DLS measurements on account of this biomodal distribution. The standard deviations in the individual measurements were comparable in size to those obtained for microbubble suspensions in previous studies [23]. They were deemed acceptable since the primary aim of these measurements was to identify any significant differences in particle size and concentration between the nanobubble suspensions that might influence the subsequent experiments.

**Table 1:** Population statistics for oxygen nanobubbles, argon nanobubbles and oxygenated water as measured by SPOS.

Suspension	Particle size (μm)				Concentration (ml)
	*Mean*	*Standard deviation*	*Mode*	*Median*	
Oxygen nanobubbles	0.86	0.70	0.63	0.66	3 x 10^7^
Argon nanobubbles	0.85	0.69	0.63	0.66	3 x 10^7^
Water	0.76	0.61	0.55	0.61	3 x 10^6^

**Fig 2:**
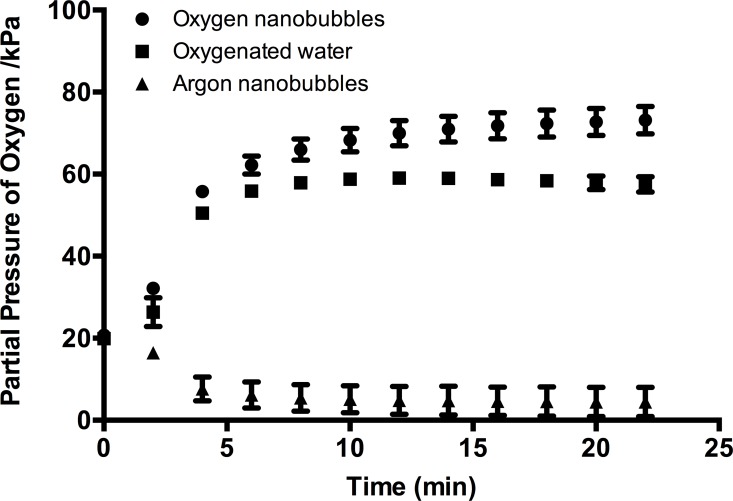
Partial pressure of oxygen (pO_2_) in water following introduction of oxygen nanobubbles (circles), argon nanobubbles (triangles) and oxygenated water (squares).I initial reading represents air-saturated water.

Fig 2 shows how the partial pressure of oxygen (pO_2_) in the suspensions varied as a function of time following injection into the monitoring system. As expected there was a negligible difference in the oxygen content of the argon nanobubble suspension compared with that of untreated water (data not shown). The oxygen-sparged water showed a rapid elevation in pO_2_ followed by a gradual decline. The oxygen nanobubble suspension showed a similar rise in pO_2_ but to a higher level and remained elevated for the duration of the experiment (25 min). Repeating the measurements at 37°C showed a small (~10%) decrease in the maximum value of pO_2_ in the oxygen nanobubble suspension. It is important to note that the monitoring system is designed to measure dissolved oxygen and therefore the values obtained for the oxygen nanobubble suspension are likely to be an underestimate, as the liquid in the closed flow loop would have quickly saturated.

**Fig 3:**
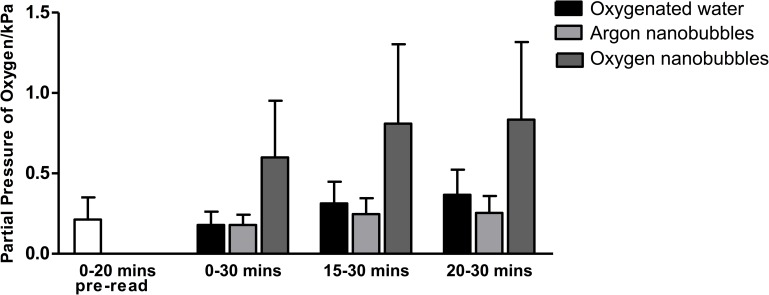
Average change in partial pressure of oxygen relative to pre-gavage value over different time periods in a mouse xenograft tumour model for human pancreatic cancer following administration of oxygen nanobubbles (dark grey), argon nanobubbles (black) and oxygenated water (light grey). (n = 8) No statistically significant differences were observed. The mean pO_2_ value recorded for the 20 min before gavage is also shown for completeness (white) (n = 24).

Fig 3 shows the corresponding *in vivo* average pO_2_ measurements made in the tumours following administration of the suspensions over different time periods. The measurements are shown relative to pre-treatment levels. The results show an apparently substantial increase in tumour pO_2_ in the 30 minute period following administration for the oxygen nanobubble treated mice relative to the other groups. The difference, however, was not found to be statistically significant. It should be noted that, in the interests of completeness, all of the acquired data (i.e. for all mice in each of the groups) were included in the analysis. These include measurements from mice for which there was no change in pO_2_ before and after administration (of which there were 2 in each group). This is the reason for the large standard deviations in the results for the group receiving the oxygen nanobubbles. The complete absence of any change in the measurement was unexpected and may have been due to the placement of the probe in a part of the tumour that was relatively poorly perfused. This is unfortunately an inherent drawback of fibre optic probe measurements.

**Fig 4:**
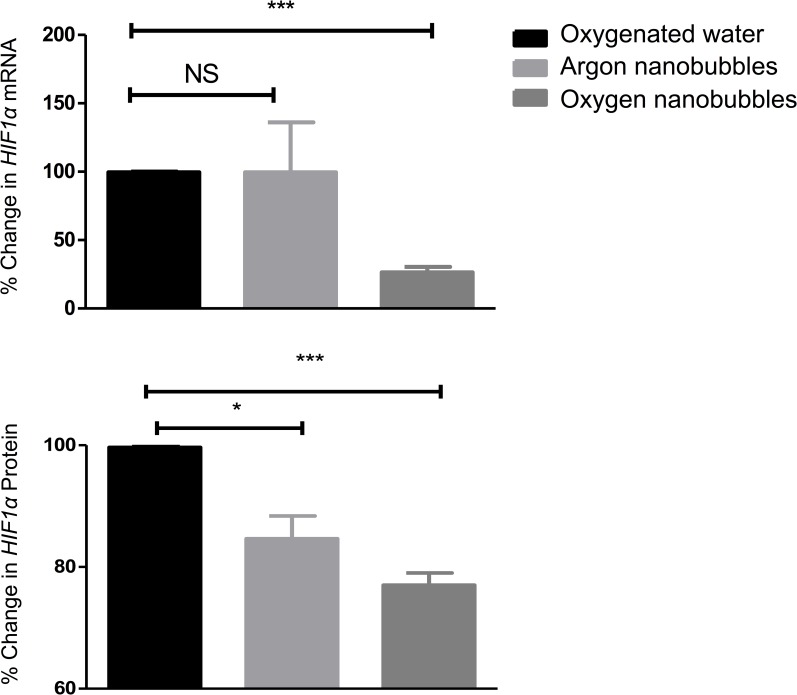
**Expression of *HIF1α* at (a) a transcriptional level and (b) a translational level in a mouse xenograft tumour model for human pancreatic cancer following administration of oxygen nanobubbles (dark grey), argon nanobubbles (light grey) and oxygenated water (black).** Animals were sacrificed 30 minutes post treatment (n = 3 per group, * p<0.05; ** p<0.01; *** p<0.001, NS = not significant).

Consequently, in order to determine whether the oxygen nanobubbles were capable of delivering oxygen to the tumour microenvironment, *HIF1*α expression was measured. Fig 4A and 4B show that animals treated orally with oxygen nanobubbles showed a reduction in *HIF1*α expression both at transcriptional (mRNA) and translational (*HIF1α* protein). Since a rapid reduction in *HIF1*α is an immediate and direct physiological consequence of increased pO_2_, these data further support our suggestion that oral administration of the oxygen nanobubbles resulted in delivery of oxygen to the tumour microenvironment. Some previous studies have shown that argon can also alter *HIF1*α expression in certain cell types *in vitro* [24]. The fact that we still observed a statistically significant decrease in HIF1α protein suggests that the effect of argon in our *in vivo* model was comparatively small. It might, however be responsible for the difference between the results for water and the argon nanobubble suspension shown in Fig 4B which was unexpected.

**Fig 5:**
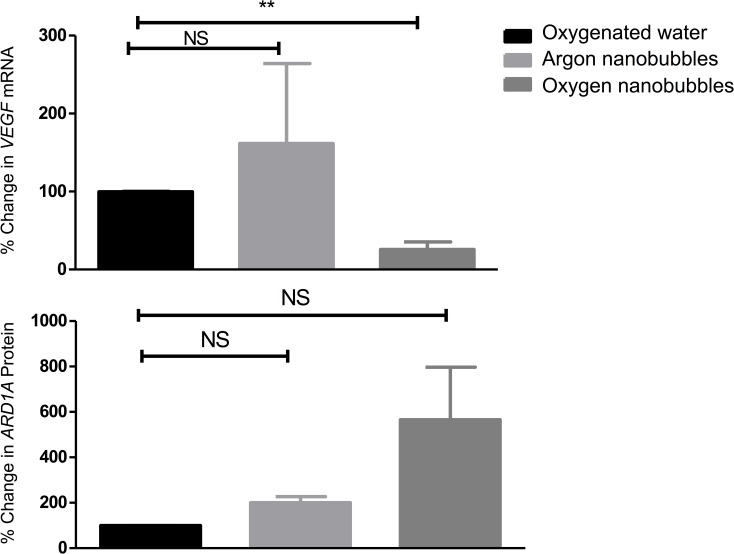
**Percentage change in expression of (a) *VEGF* mRNA and (b) ARD1A protein in in a mouse xenograft tumour model for human pancreatic cancer following administration of oxygen nanobubbles (dark grey), argon nanobubbles (light grey) and oxygenated water (black).** Data are shown relative to measurements for oxygenated water. Animals were sacrificed 30 minutes post treatment (n = 3 per group, * p<0.05; ** p<0.01; *** p<0.001, NS = not significant).

Further indication of oxygen-induced physiological changes in the tumours is provided in Fig 5. Panel (a) shows that *VEGF* expression was significantly reduced in the tumours treated with the oxygen nanobubble suspension. Panel (b) shows that there was also a large increase in the expression of ARD1A protein in this group; although due to one measurement in the oxygen nanobubble group being exceptionally high, the corresponding standard deviation means that the result is not statistically significant. Neither oxygenated water nor the argon nanobubbles produced any statistically significant changes. ARD1A-mediated acetylation of HIF-1α at Lys532 under normoxic conditions enhances binding of Von Hippel Lindau (VHL) protein, leading to increased ubiquitination and degradation of HIF-1α and down-regulation of HIF-1α target genes [25].

In terms of therapeutic relevance, we have recently demonstrated that a similar reduction in *HIF1*α expression produced by injected oxygen microbubbles did lead to a significant improvement in treatment efficacy with sonodynamic therapy in the same tumour model [14]. Similarly, previous studies have indicated that the response to radiation therapy is highly dependent upon tumour oxygenation with an increase from 2.5 mmHg (0.33 kPa) to 6 mmHg (0.8 kPa) significantly affecting loco-regional tumour control in advanced squamous cell head and neck carcinoma [26].

As above, the ability to reduce hypoxia via an orally delivered agent could have considerable advantages in terms of cost, convenience and patient acceptability/compliance. Clearly, however, there are several important questions to answer: First, the results from previous studies involving peritoneal infusions of microbubbles [13] or oxygen-carrying liquid droplets suggest that gas exchange between the peritoneal cavity and blood pool occurs leading to elevated blood pO_2_. It seems likely that a similar process is occurring in the case of oral delivery although further testing is needed to determine whether any nanobubbles pass into the blood intact. This is important because if oxygen is present in the circulation in encapsulated form then the total amount available will be greater than that due to haemoglobin and plasma saturation alone. This is particularly relevant given the significantly larger distances over which oxygen would need to be transported in a human. In this respect, it is possible that oxygen distribution could be enhanced through the use of a physical stimulus such as ultrasound, which has been shown to improve penetration of gas carrying nanoparticles into tumours in other studies (27).

Of perhaps even greater importance is the need to determine whether the rise in blood oxygen content is sufficient and/or sufficiently sustained to present a risk of oxygen toxicity. One difference between this approach and hyperbaric treatments is that the saturation concentration of oxygen in tissue is not being modified because neither temperature nor pressure is altered. Rather, the quantity of oxygen available is increased and there should therefore be selective delivery to regions where the oxygen concentration is lower. In our treatments, we did not notice any overt adverse effects on the animals following gavage and the absence of adverse effects reported in previous studies of peritoneal administration of microbubbles is encouraging [13], but this undoubtedly requires further investigation.

## Conclusions

In summary, there is a need for improved methods to reduce tumour hypoxia in order to increase the efficacy of current cancer therapies. Oral delivery of oxygen loaded nanobubbles could potentially provide a convenient delivery vehicle to enable the transient oxygenation of hypoxic tumours that would be compatible with existing cancer treatment regimes. In this study, direct measurements of tumour oxygenation using a fibre optic probe, were characterised by very large standard deviations and did not show a statistically significant difference between treatment groups. Examination of *HIF1*α and *VEGF* expression, however, indicated that oxygen nanobubbles could produce a statistically significant reduction to a level that has been associated with improved treatment outcomes in previous studies using the same pancreatic tumour model. Further investigation of the mechanisms by which gas transfer occurs within the circulation and tissue is required to determine whether or not the effects observed would be replicated in humans and whether there are any adverse side effects.

## Supporting Information

S1 Fig (a) Plot of tumour oxygenation as a function of tumour growth for the ectopic BxPC3 tumour model used in this study. (b) Plot of tumour volume against time for the tumours measured in (a). Tumour oxygen (pO_2_mmHg) was measured via an OxyLite oxygen electrode and readings converted to % oxygen (n = 5).
(TIFF)

S2 Fig (a) Representative example of measured tumour pO_2_ recorded every min for 30 min after oral gavage of either (i) oxygen nanobubbles (solid black line) (ii) argon nanobubbles (dotted black line) and (iii) oxygenated water (solid grey line) (n = 8).
(TIFF)
