# Supplementary figures and images for: Reducing Tumour Hypoxia via Oral Administration of Oxygen Nanobubbles
Source: PLoS One. 2016 Dec 30;11(12):e0168088. doi: 10.1371/journal.pone.0168088 (PMC5201233; doi:10.1371/journal.pone.0168088)

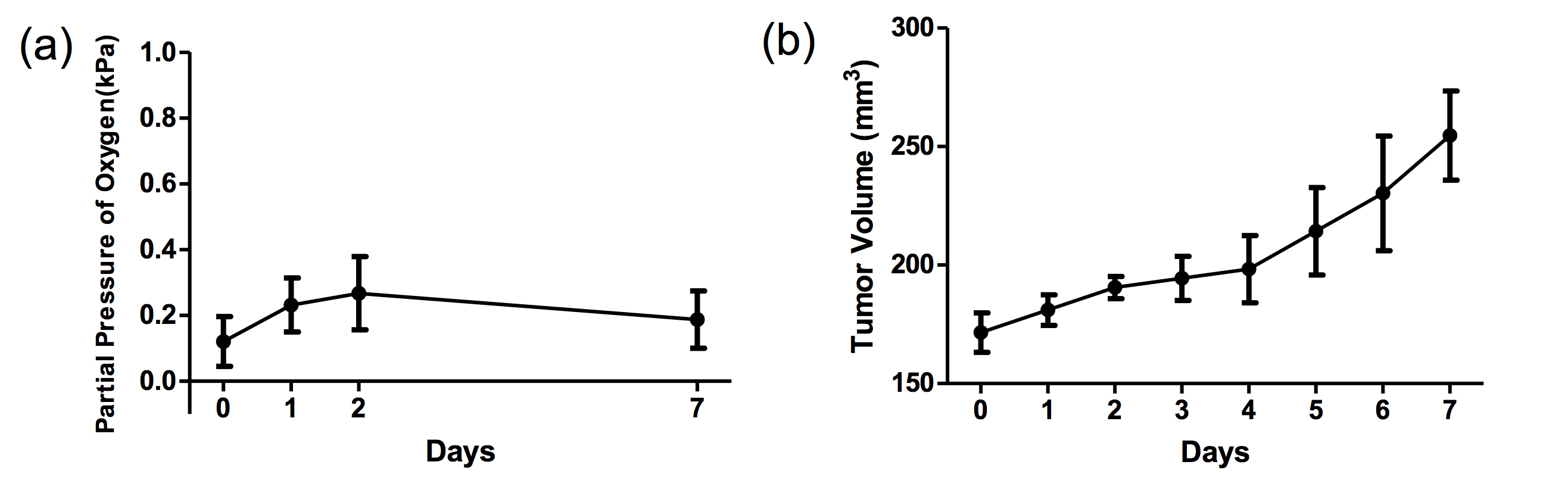

Supplement: S1 Fig — (a) Plot of tumour oxygenation as a function of tumour growth for the ectopic BxPC3 tumour model used in this study. (b) Plot of tumour volume against time for the tumours measured in (a). Tumour oxygen (pO2mmHg) was measured via an OxyLite oxygen electrode and readings converted to % oxygen (n = 5). (TIFF) [file pone.0168088.s001.tiff]

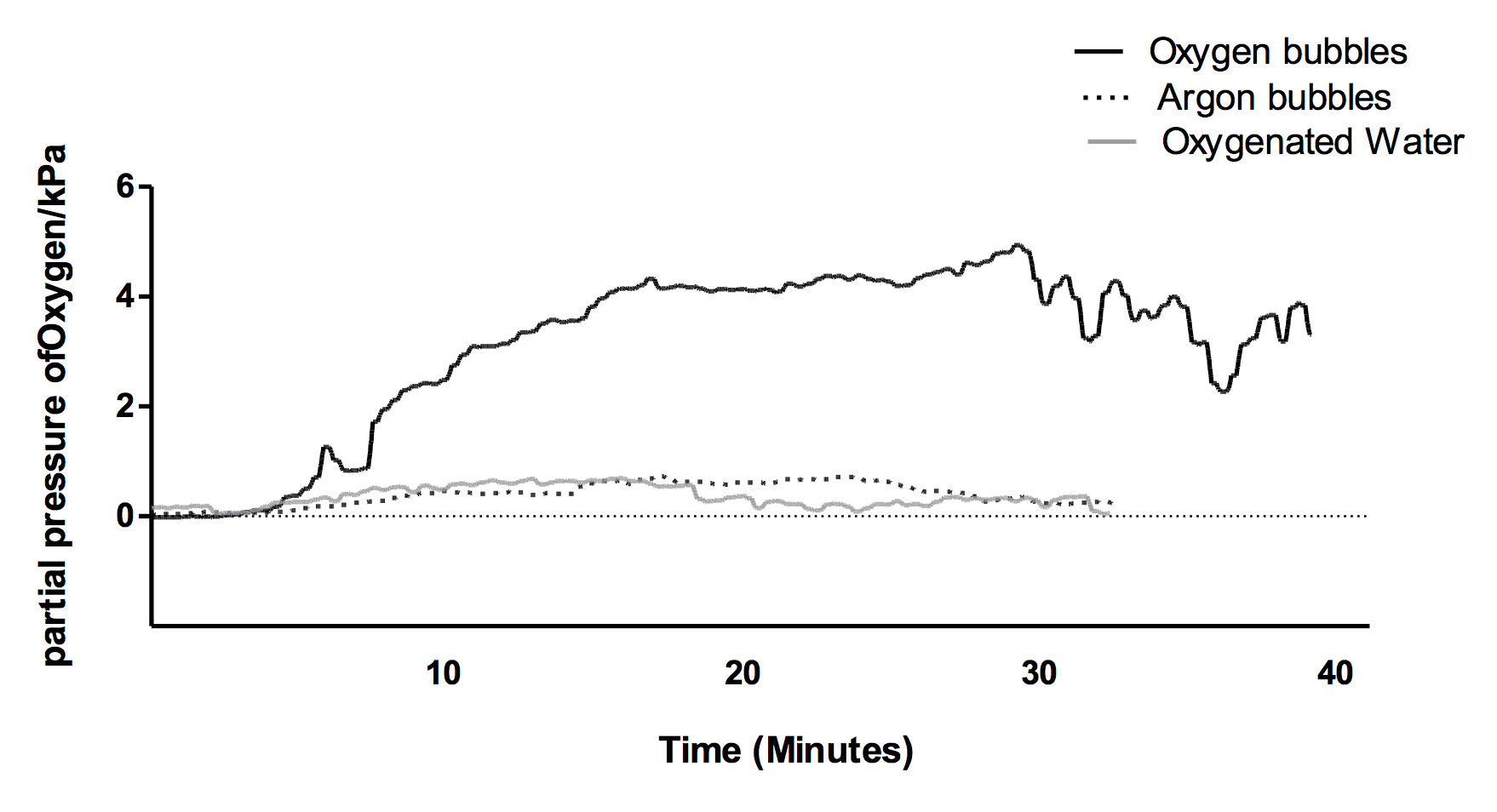

Supplement: S2 Fig — (a) Representative example of measured tumour pO2 recorded every min for 30 min after oral gavage of either (i) oxygen nanobubbles (solid black line) (ii) argon nanobubbles (dotted black line) and (iii) oxygenated water (solid grey line) (n = 8). (TIFF) [file pone.0168088.s002.tiff]
